# Study on Boundary Layer and Surface Hardness of Carbon Black in Natural Rubber Using Atomic Force Microscopy

**DOI:** 10.3390/polym14214642

**Published:** 2022-10-31

**Authors:** Jian Chen, Mao-Yuan Hu, Long Qing, Ping Liu, Lin Li, Rui Li, Cheng-Xi Yue, Jarrn-Horng Lin

**Affiliations:** 1 Department of Materials Science and Engineering, Sichuan University of Science and Engineering, Zigong 643000, China; 2 Sichuan Province Key Laboratory for Corrosion and Protection of Material, Sichuan University of Science and Engineering, Zigong 643000, China; 3 Department of Materials Science, National University of Tainan, 33, Sec. 2, Shu-lin St., Tainan 70005, Taiwan

**Keywords:** carbon black, natural rubber, surface hardness, bonded rubber, atomic force microscopy

## Abstract

The mechanical properties and wear resistance of carbon black/natural rubber (CB/NR) composites are significantly influenced by the degree of CB dispersion in rubber. Here, we present a novel reinforcement theory using atomic force microscopy (AFM) to quantify the adhesive thickness of rubber molecules around the CB particles as well as the height, area, and volume in NR. The thickness of the bonded rubber (BR) was found to vary between 3 and 7 nm depending on the values of the nitrogen surface area (NSA) for CB. This indicates that a higher BR content is a result of a higher CB NSA with a smaller particle size, showing a higher number of active positions to anchor rubber molecules. The nanoindentation of AFM was used to determine the surface hardness of CB in NR; the value decreases with increasing BR height. In this study, we demonstrate a well-defined reinforcement mechanism of CB in NR with the factors of BR, surface hardness, 100%/300% modulus, and tensile strength.

## 1. Introduction

Filler-reinforced rubbers have been widely used in a variety of applications, particularly in the tire industry for several decades. However, their reinforcement mechanisms and filler-rubber-bounded structures have long been studied and are still undefined [1,2,3]. The improved mechanical properties of rubber compounds for tire applications, e.g., wear resistance, rolling resistance, and wet traction are primarily determined by the type of filler, carbon black (CB), silica, or other species. CB is commonly used as a reinforced filler in rubbers to improve their mechanical properties. Meanwhile, adding CB to the rubber matrix can improve its elastic modulus, fracture strength, and wear resistance significantly [4,5]. As a result, various CBs, e.g., N115, N330, N550, N660, and others, have been used to improve the mechanical properties of various rubbers. CB/natural rubber (NR) has been widely used in a wide range of practical applications, and numerous theories exist about CB improving rubber wear resistance. Normally, the theory of bound rubber is commonly used to describe the interaction between CB and rubber molecules [6,7]. Conceptual models of bound rubber have generally focused on the insoluble rubber phase as the adhesive center between CB and rubber [6,7]. Previous research has demonstrated that bound rubber is a useful index for understanding CB dispersion in rubber matrixes for mechanical reinforcements. The reinforcing effect was found to be closely related to the CB primary particle size, structures, and activities [8,9]. Many studies have shown that when the specific surface area of CB is greater than 50 m^2^ g^−1^ and the particle size is less than 50 nm, it is an active reinforcing filler for rubber [10].

Previously, the reinforcing mechanism in CB/rubber systems was primarily reported using elastic modulus predictions, stress–strain simulations, and considerations for the strengthening mechanism [11,12,13]. Saowapark et al. [11] established a link between the amplitude dependence of the filled rubber elastic deformation and the structural properties of the filler. Huber et al. [12] described the mechanical deformation behavior of rubbers filled with rigid filler particles. Liang et al. [13] revealed the relationship between the local nanoscale stress distribution and macroscopic tensile properties. Robertson et al. [14] recently reported that a strong reinforcement of the filler–elastomer system results in the formation of a glassy layer covering the filler surface. Kohjiya et al. [15] observed CB networks in the rubbery matrix using a three-dimensional transmission electron microscopy (3D-TEM) skeletonization image. The accomplishments listed above were primarily focused on using the physical/chemical combination network of the microstructure of the CB surface and the rubber polymer chain reinforcement to clearly explain the improvement of rubber tear resistance and modulus. However, no accepted theory exists that fully explains the CB reinforcement mechanism, particularly the spatial dispersion and interactions of CB in the rubber matrix. A simple and logical explanation for how CB-reinforced rubber can improve the wear resistance and service life of rubber is unknown.

The bonded rubber around the CB must be studied in order to investigate the CB strengthening mechanism in rubber. Until now, the dispersion of CB in rubbers has been primarily studied using electron and atomic force microscopes (AFM) [16,17,18,19,20,21,22,23,24]. Le Diagon et al. [20] investigated the dispersion of fillers in a composite using AFM. Using an AFM nanomechanical mapping technique, Wang et al. [21] discussed the topography, modulus, and adhesive energy distribution maps of the resulting composites in terms of carbon nanotube loading. Vera-Agullo et al. [22] proposed studying the microstructure of filled rubber using optical microscopy and AFM. Xu et al. [23] employed AFM to characterize the structure and morphology of CB. A large number of works have presented the AFM results of filled polymer structures in various ways [24]. Although these reported AFM results can reveal several filler–rubber reinforcing behaviors, e.g., morphology and filler spatial dispersion, there is, however, no comparison has been made between different types of CB when rubber using AFM to determine the spatial properties of the CB-filled rubber.

The thickness of bonded rubber formed by different types of CB was investigated using AFM and TEM in this study. Furthermore, nanoindentation was used to determine the hardness of various CBs in rubber. More importantly, our approach has the potential to provide new insights into the microscopic reinforcement mechanism of CB/NR composites.

## 2. Experimental

### 2.1. Materials

Zinc oxide (ZnO), stearic acid (SA), sulfur (S), mercaptobenzothiazole disulfide (MBTS), which are purchased from Merck, Kenilworth, NJ, USA. CB (N115, N330, N550, N660), and NR were supplied from the Carbon Black Research and Design Institute, Zigong, Sichuan, China.

### 2.2. Preparation of the CB/NR Composites

Rubber composites were prepared by the mill-mixing method according to ASTM D 3192: 2005, as shown in Table 1. For a typical run, the roller temperature was kept at 70 °C and the total run time was maintained at 17 min. NR was placed on a two-roller mill (model zg-200dr, China Two Roll Mill, Shanghai, China) and masticated for 2 min. Sequentially, 3 phr (parts per hundred of rubber) SA, 2.5 phr S, 0.6 phr MBTS, 5 phr commercial microscale ZnO (denoted as c-ZnO), and 50 phr N115 were added. The (N330/NR, N550/NR, and N660/NR) composites were prepared using the same procedure. The structural properties of CB are listed in Table 2. Sulfur (normally in the form of crown-like structure-S_8_) is used as a cross-linking bridge between CB and NR, a process known as vulcanization. During the vulcanization process, ZnO and SA are used as activators, and the MBTS function is prompted to shorten the vulcanization time.

Tensile measurements were conducted based on the ASTM D412 Standard Test Method with a type C dumbbell die. Three specimens were tested for each sample using an Instron Model 3400. The tensile rate was 500 mm/min, the initial grip distance was 60 mm. A video system was used to measure strain to determine the 100/300% modulus.

### 2.3. Sampling and TEM/AFM Characterization

The samples were cut into 200 μm × 200 μm sections of about 50 nm thickness using an ultra-microtome cryostat (Leica EM UC7, Leica Microsystems Trading Co., Ltd., Shanghai, China) at −30 °C to obtain a smooth surface, and they were subjected to high-vacuum AFM (E-SWEEP, Hitachi High-Tech Science Co., Ltd., Tokyo, Japan). All of the AFM samples were tested at 25 °C. The nanoindentation hardness of all samples was measured using a nano-indenter (Anton Paar, UNHT, Torino, Italy). For a typical test, the loading speed remained constant at 30 s with a force of 0.8 Nm and a 5 s delay at peak load with a 30 s unloading time.

**Scheme 1:**
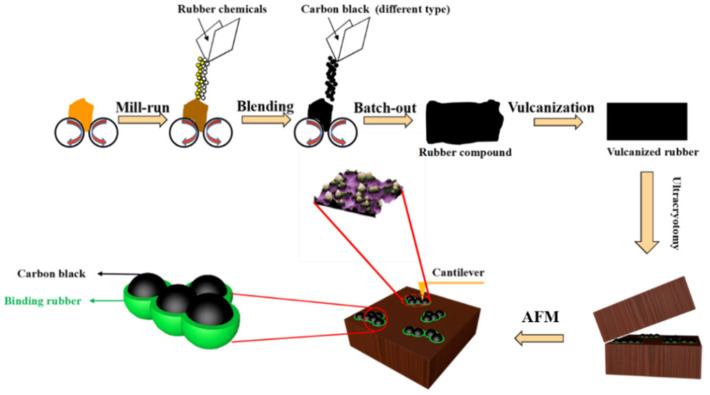
Preparation of CB/NR composites and their structural samples used in AFM/TEM measurements.

The microscopic and macroscopic morphologies of CB were observed with TEM (FESEM, Inspect F50, FEI Company, Eindhoven, The Netherlands). The structural characterization and dispersion of CB in the CB/NR composites were evaluated, and the protruding height of the CB aggregate in the rubber was assigned as the bonded rubber [13]. The preparation process of all the samples for AFM/TEM measurements is illustrated in Scheme 1.

## 3. Results and Discussion

**Figure 1:**
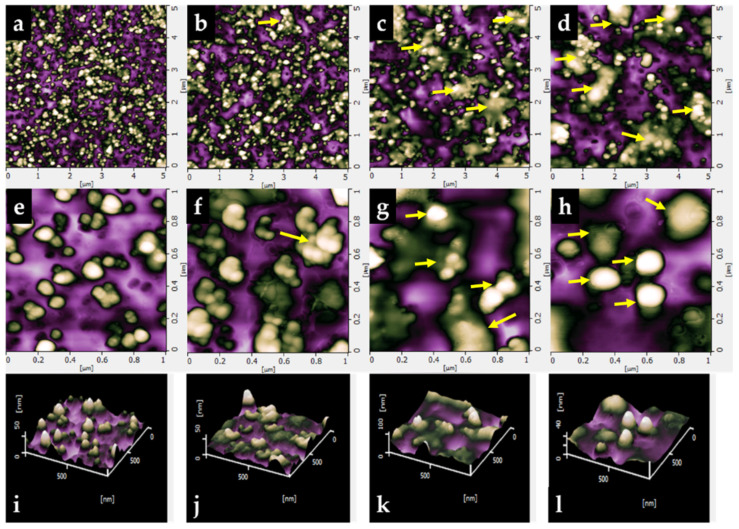
AFM micrographs of (**a**) N115/NR, (**b**) N330/NR, (**c**) N550/NR, and (**d**) N660/NR, (**e**–**h**) are the enlarged area AFM images of (**a**–**d**), and (**i**–**l**) are the three-dimensional surface morphologies of the corresponding ranges.

AFM measurements for four types of CB (N115, N330, N550, and N660) in NR are shown in Figure 1a–l. The black and white parts represent CB aggregates or NR-covered aggregates of CB, while the purple regions represent the non-CB-reinforced zone. Apparently, in Figure 1a–d, the N115/NR system (Figure 1a) shows the highest CB/NR dispersion with a dimension of 5 μm × 5 μm. The CB/NR dispersion status decreases in the order of N115/NR, N330/NR, N550/NR, and N660/NR. The enlarged areas (1 μm × 1 μm) of the four CB/NR systems are presented in Figure 1e–h. It is clear to note that while the NSA or STSA value of CB decreases, the obvious isolated particles and chain-like agglomerate are distributed in the AFM micrographs, as shown in Figure 1b–d (see yellow arrow) or enlarged parts of Figure 1f–h (see yellow arrow). This shows that the key active sites of CB are related to the surface structures rather than the particle packing styles, which are typically described with different OAN values. Figure 1i–l depict the 3D surface profiles of all CB/NR systems, indicating that the nanoparticles of N115 (Figure 1i) were well covered and dispersed within the NR molecular chain. Because of their lower NSA values, the other three CB/NR samples have more agglomerate-like structures, as shown in Figure (Figure 1j–l).

**Figure 2:**
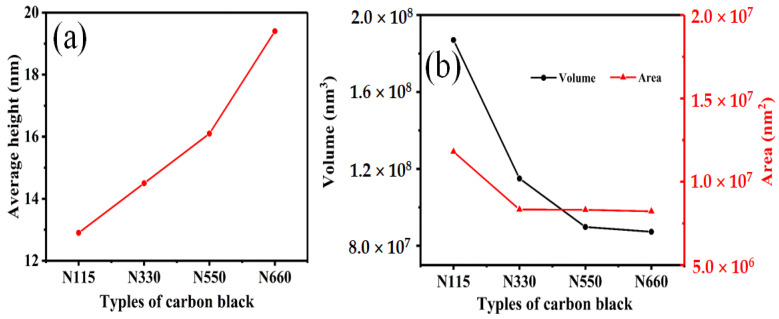
(**a**) Average protruding heights of N115/NR, N330/NR, N550/NR, and N660/NR in the area of 1000 nm × 1000 nm. (**b**) The extruded volume and area of N115/NR, N330/NR, N550/NR, and N660/NR in the area of 1000 nm × 1000 nm.

**Figure 3:**
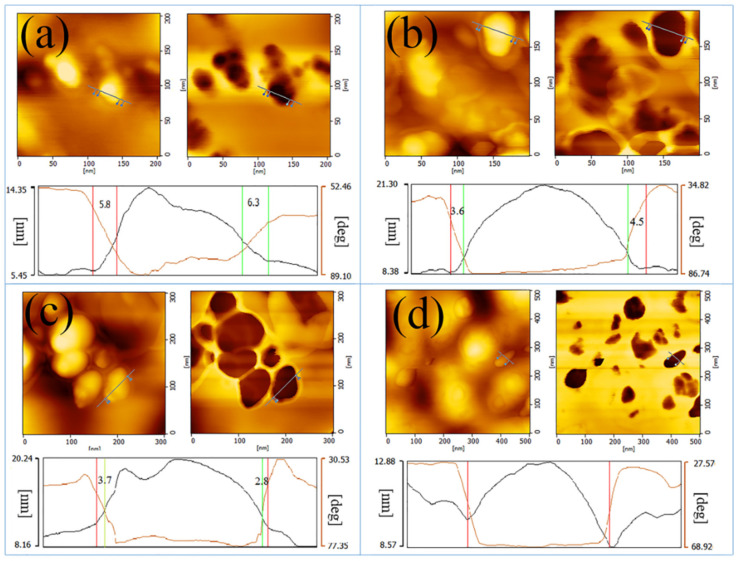
AFM height and phase images of the various CB in NR (**a**) N115, (**b**) N330, (**c**) N550, and (**d**) N660.

Figure 2 also depicts the average volume and area of all CB/NR systems. As shown in Figure 2a, the average protruding heights of N115/NR, N330/NR, N550/NR, and N660/NR samples are 12.9, 14.5, 16.1, and 19.4 nm, respectively. This demonstrates that the height of the protrusion increased as the particle size of CB increased (as shown in Table 2). The extruded volume and area of N115, N330, N550, and N660 in NR were also investigated, as illustrated in Figure 2b. The extruded volumes of N115, N330, N550, and N660 were 1.87 × 10^8^, 1.15 × 10^8^, 8.98 × 10^7^, and 8.73 × 10^7^ nm^3^, respectively. This indicates that a smaller CB particle can strongly interact with NR molecules, resulting in a higher CB/NR dispersion, which also increases the protruding volume. Similarly, the extruded area of N115, N330, N550, and N660 were 1.18 × 10^7^, 8.33 × 10^6^, 8.31 × 10^6^, and 8.22 × 10^6^ nm^2^, respectively. This demonstrates that decreasing the CB particle size can improve dispersion in the NR matrix. Figure 3 depicts the morphologies, phase diagrams, and section profiles of the four types of CB analyzed by AFM. 

The boundary between N115 and the rubber matrix is hazy and blurry (Figure 3a), indicating that N115 particles interact strongly with NR molecules. As shown in Figure 3b–d, the boundary between CB and NR is more distinct owing to the larger particle sizes and lower NSA values. The AFM phase diagrams of all CB/NR samples reveal that decreasing the primary particle size of CB improved microscopic dispersion, indicating that CB with smaller particle sizes had better reinforcing behaviors in NR. The wear resistance of CB-filled rubber was also reported to be similar due to a higher micro-dispersion of CB in rubber. The bonded rubber thicknesses for the four samples were estimated using the results shown in Figure 3. To accurately analyze the positions of the structures, AFM analysis software was used to mark the same line at the corresponding positions of the topography and phase diagrams, and the CB edge points of the line in the topography were marked with points designated by the two marks inside the two graphs. Simultaneously, the position of the rubber was marked on the phase diagram’s middle line by the two points outside the line, indicating that the brown structure was between the CB aggregate and the natural rubber. The phase value of the inner marker remained essentially constant in the section contour curve, while the height value of the area from the inner marker to the outer marker gradually decreased. Thus, the hardness of CB/NR was measured in the brown area, which also indicated the bound rubber phase. Figure 3a–d show that the bonded rubber of N115 was as thick as 7 nm, N330 and N550 were 4 and 3 nm, respectively, and N660 was barely visible. This is because N115 had a larger specific surface area which allowed for more rubber anchor sites [22]. The glassy state of the bond rubber attached to the CB particle sizes, as well as the various bonded rubbers formed by the interaction of different CB particle sizes and rubber molecules, would accompany the change in hardness.

**Figure 4:**
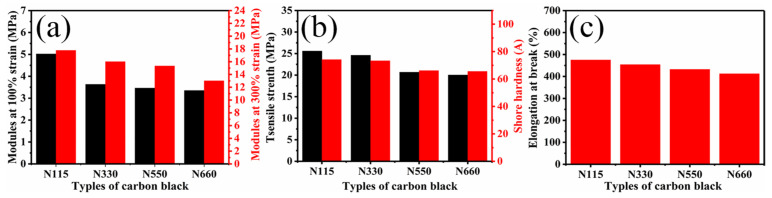
Mechanical properties of the CB (N115, N300, N550, and N660)-filled NR samples. (**a**) The 100% and 300% modulus values of the various CB-filled NR. (**b**) Tensile strength and hardness of the rubber filled with the various CB-filled NR. (**c**) Elongation at break of the rubber filled with the various CB-filled NR.

**Figure 5:**
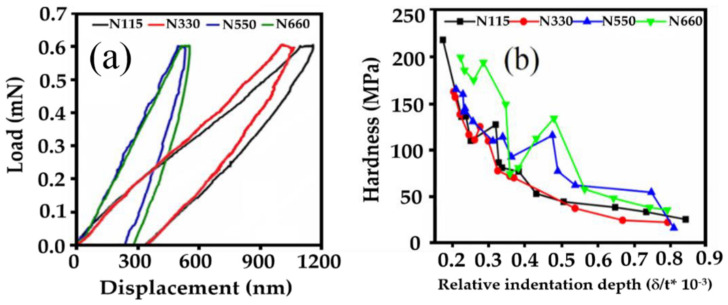
(**a**) Typical force–displacement curves for CB/NR. (**b**) Variation in the hardness of the four different CB/NR composites with relative indentation depth.

Figure 4 depicts the physical properties of the CB/NR composites. The modulus values of the CB/NR composites at 100% and 300% appear to have gradually decreased as the CB particle size increased. This was due to the smaller spacing between the CB aggregates with small particle sizes, as well as the more uniform adhesive distribution, which played a better role in fixing the CB [25,26]. This results in a higher wear resistance of CB and rubber. The tensile strength and hardness of CB/NR composites decreased progressively as the CB particle size increased. (Figure 4b) [27,28,29,30]. Figure 4c shows that N115/NR had the greatest elongation at break, owing to the thicker bond rubber generated by N115. The typical load–displacement curves of all CB/NR composites are shown in Figure 5a. 

The displacement of the probe in the N660/NR composite was the smallest when the same load was applied to the surfaces of the rubber composites, indicating that the hardness of the N660/NR composite was higher than that of the other composites. Recent studies have discovered that the interphase between nanoparticles and the rubber matrix has an inhomogeneous elastic modulus and plastic deformation [31,32]. This interphase heterogeneity is important in the macroscopic mechanical properties of CB-reinforced rubbers. The elastic modulus and plastic deformation of spherical CB and its surrounding bound rubber in NR are predicted using a comprehensive micromechanical framework developed in this study. A loading force of 0.8 mN was chosen after a series of loading–unloading process tests at an indentation point using the basic standard method. The elastic modulus (E_s_) of all CB/NR composites was calculated using the following Equation (1), where E_r_ is the elastic modulus of pattern loss, E_i_ is the elastic modulus of indenter, V_i_ is the Poisson’s ratio of pattern materials, and Es and Vs are the elasticity and Poisson’s ratio of pattern material, respectively. According to Equation (1), the hardness of N115/NR, N330/NR, N550/NR, and N660/NR are 2.37, 2.75, 7.24, and 19.72 GPa, respectively. The N660/NR had the highest hardness, reaching 19.72 Gpa, because the thickness of the bonded rubber surrounding the surface of CB-N115, N330, and N550 gradually decreased, and the bonded rubber was a combination of rubber polymer chains and CB, with some elasticity. As our findings show, the N660/NR had a higher hardness because there was no existing bonding rubber. Therefore, the AFM test probe made direct contact with the N660′s surface. Accordingly, N115 had the smallest particle size of all samples, resulting in the highest bound rubber content and the lowest hardness.

E_r_^−1^ = 1 − V_i_^2^/Ei + 1 − V_s_^2^/E_s_ (1)

C_IT_ = h_2_ − h_1_/h_1_ × 100% (2)

Furthermore, the creep of the CB/NR composites was also assessed. The common macroscale method to study the creep of the samples is to measure the displacement under a fixed load, and by using Equation (2), the microscale status was estimated, where h_1_ is the indentation depth at the maximum load in overload loading, and h_2_ is the indentation depth at the end of the retaining load. The C_IT_ values for N115, N330, N550, and N660 were 5.75%, 5.65%, 7.46% and 8.22%, respectively. It can be seen that with the increasing particle size of CB, the composite was more prone to creep. This could be related to the content of the bonded rubber around the CB. Aside from the N660/NR sample (see Figure 5b), the samples exhibited similar hardness behaviors at shallow indentation depths. The hardness of the four composites gradually dispersed as the indentation depth increased.

**Figure 6:**
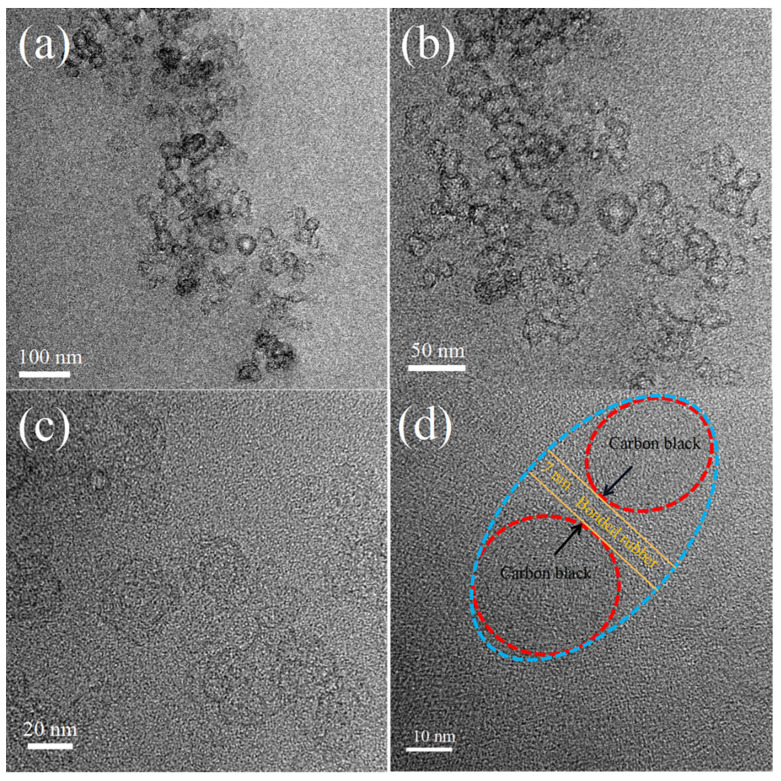
The HRTEM images of N115/NR nanocomposite with different magnifications, (**a**) 100 nm, (**b**) 50 nm, (**c**) 20 nm, and (**d**) 10 nm for the scale bar.

The TEM photographs of the N115/NR composite are shown in Figure 6a–d, the darker part belongs to the nanoparticles of N115, while the light-colored area is the rubber matrix of NR.

A clearer outline of the CB particles can be seen in Figure 6a–d, and the darker the color, the more overlapping the CB particles. Figure 6d shows that the binding bonded rubber around the CB is approximately 7 nm, which supports our findings in the AFM measurements shown in Figure 3.

## 4. Conclusions

Our findings suggest a novel approach to understanding the nanoscale BR and reinforcing mechanism of automobile tires. CB with higher specific surface areas provides more anchor sites to bind with rubber. As a result, the bound rubber thickness of N550, N330, and N115-based NR samples increased from 3 to 7 nm. However, due to a lower NSA value, the N660-based NR sample showed no existence of bound rubber. The results of our AFM/TEM measurements can provide new insights into the clear interaction of reinforced filler-CB and rubber molecules.

## Figures and Tables

**Table 1:** Formula of the CB-filled NR compounds.

Component	Composition (phr)
NR	100
Sulfur	2.5
Steric acid	3
c-ZnO	5
CB (N115, N330, N550, N660)	50
MBTS	0.6

**Table 2:** Structural properties of CB used in this study.

	Item	Particle Size (nm)	NSA (m^2^ g^−1^)	STSA (m^2^ g^−1^)	Oil Adsorption Number (OAN) (mg g^−1^)	Ash (%)
CB						
**N115**		16 ± 2	140	124	115	0.3
**N330**		32 ± 4	78	75	100	0.4
**N550**		47 ± 8	40	36	121	0.6
**N660**		63 ± 6	34	29	90	0.6

**Note**: The particle size of CB was measured by TEM, and the nitrogen surface area (NSA) was determined by the nitrogen adsorption/desorption profiles at −196 °C from P/P_0_ = 0.05 to 0.30 according to the Brunauer–Emmett–Teller (BET) method based on ASTM D6556. STSA means statistical thickness surface area, which represents the external surface area of CB according to ASTM D6556. Oil adsorption number (OAN) stands for the particle packing style of CB according to ASTM D1765. Ash content was measured based on the method in ASTM D1506-15.

## Data Availability

Not applicable.
